# The Role of Sirt1 in Epileptogenesis

**DOI:** 10.1523/ENEURO.0301-16.2017

**Published:** 2017-02-10

**Authors:** Alicia M. Hall, Gary P. Brennan, Tiffany M. Nguyen, Akanksha Singh-Taylor, Hyun-Seung Mun, Mary J. Sargious, Tallie Z. Baram

**Affiliations:** 1 Department of Pediatrics, University of California, Irvine, Irvine, California 92697; 2 Department of Anatomy and Neurobiology, University of California, Irvine, Irvine, California 92697

**Keywords:** epigenetics, epilepsy, epileptogenesis, intervention, metabolic stress, sirtuins

## Abstract

The mechanisms by which brain insults lead to subsequent epilepsy remain unclear. Insults, including trauma, stroke, tumors, infections, and long seizures [status epilepticus (SE)], create a neuronal state of increased metabolic demand or decreased energy supply. Neurons express molecules that monitor their metabolic state, including sirtuins (Sirts). Sirtuins deacetylate cytoplasmic proteins and nuclear histones, and their epigenetic modulation of the chromatin governs the expression of many genes, influencing neuronal properties. Thus, sirtuins are poised to enduringly modulate neuronal properties following SE, potentially contributing to epileptogenesis, a hypothesis supported by the epilepsy-attenuating effects of blocking a downstream target of Sirt1, Neuron-Restrictive Silencer Factor (NRSF) also know as REST (RE1-Silencing Transcription factor). Here we used an adult male rat model of epileptogenesis provoked by kainic acid–induced SE (KA-SE). We assessed KA-SE-provoked Sirt1 activity, infused a Sirt1 inhibitor (EX-527) after KA-SE, and examined for epileptogenesis using continuous digital video–EEG. Sirt1 activity, measured using chromatin immunoprecipitation for Sirt1 binding at a target gene, increased rapidly after SE. *Post hoc* infusion of the Sirt1 inhibitor prevented Sirt1-mediated repression of a target gene. Blocking Sirt1 activity transiently after KA-SE did not significantly influence the time- course and all of the parameters of epilepsy development. Specifically, latency to first seizure and seizure number, duration, and severity (using the Racine scale and EEG measures) as well as the frequency and duration of interictal spike series, were all unchanged. KA-SE provoked a robust inflammatory response and modest cell loss, yet neither was altered by blocking Sirt1. In conclusion, blocking Sirt1 activity after KA-SE does not abrogate epilepsy development, suggesting that the mechanisms of such acquired epileptogenesis are independent of Sirt1 function.

## Significance Statement

Epilepsy is the third most common chronic brain disorder. It is often triggered by insults to the brain such as trauma, stroke, and long seizures. These insults alter neuronal metabolism, which is then sensed by sirtuins. Here, we examine the role of Sirt1 in the mechanism of insult-induced epilepsy development. We effectively blocked, using *post hoc* intervention, the rapid increase in Sirt1 activity. Notably, this intervention did not abrogate insult-provoked epileptogenesis or the associated inflammatory response and modest cell loss. These findings suggest that Sirt1 activation is not required for KA-SE. Epileptogenesis or downstream actions of this potent transcriptional regulator play complex and perhaps suggest opposing roles in epileptogenesis.

## Introduction

Epilepsy is among the most common chronic neurological disorders, affecting >1% of the population. Brain insults, including early-life or adult long seizures [status epilepticus (SE)], stroke, traumatic brain injury (TBI), and infection, commonly precede epilepsy in humans and generate the disorder in animal models. However, the mechanisms in which these insults increase the risk for developing epilepsy are unclear. Insults change neuronal network properties (Engel et al., 2013; Goldberg and Coulter, 2013; Lillis et al., 2015). Network changes can result from cell death-induced circuit reorganization, which plays a role in a number of, but not all, models of insult-induced epilepsy (Toth et al., 1998; Dingledine et al., 2014). Notably, neuronal characteristics are changed drastically in epileptic tissue from humans and rodents (Bender et al., 2003; Bernard et al., 2004; Becker et al., 2008; Hudry et al., 2012; Surges et al., 2012; Lillis et al., 2015; Gast et al., 2016). These cellular changes are driven, at least partly, by changes in gene expression (Brooks-Kayal et al., 2009; McClelland et al., 2014; Rossignol et al., 2014). Further delineation is needed on how insults provoke the often large-scale changes in gene expression and how these changes persist.

Insults that promote epilepsy such as SE, TBI, and infection are often metabolically demanding (Duffy et al., 1975; Fujikawa et al., 1988; Babikian et al., 2010; Carmody and Brennan, 2010; Rowley and Patel, 2013; Rho, 2015; Liang and Patel, 2016). Metabolic demand/cell stress activates mechanisms of gene expression that change neuronal function (Garriga-Canut et al., 2006). Silent information regulator 2 proteins [sirtuins (Sirts)] bridge metabolism and gene expression. Sirtuins are a class III histone deacetylase that require NAD^+^ for their enzymatic activity as a protein deacetylase (Blander and Guarente, 2004; Herskovits and Guarente, 2014). Therefore, their activation is linked to the energy status of the cell through the NAD^+^/NADH ratio (Canto and Uwerx, 2012; Srivastava, 2016). Sirt1 is located predominantly in the nucleus of neurons. Consequently, Sirt1 responds quickly to cellular conditions of energy demand and deacetylates histones to affect the state of the chromatin and, hence, gene transcription (Canto and Uwerx, 2012; Srivastava, 2016).

The neuroprotective effects seen with caloric restriction are thought to involve Sirt1-mediated deacetylation (Gräff et al., 2013; Libert and Guarente, 2013), leading to consideration of resveratrol to treat neurodegeneration in multiple models (Kim et al., 2007; Wu et al., 2009; Mancuso et al., 2014; Li et al., 2015). However, recent literature has insinuated that the role of Sirt1 in cellular function and disease modification is far more complex and nuanced (Ng and Tang, 2013). This may be a result of the numerous and potentially conflicting actions of Sirt1, including regulation of metabolism, apoptosis, autophagy, and mitochondrial function.

Sirt1 is upregulated in epilepsy patients (Chen et al., 2013) and after a proepileptic insult in rat models (Chen et al., 2013; Wang et al., 2015; Brennan et al., 2016), yet a role for Sirt1 in seizure protection has also recently been suggested (Wang et al., 2016). Sirt1 inhibition was protective in some Huntington’s disease models (Smith et al., 2014), and overexpression of Sirt1 induced a reference memory deficit (Kakefuda et al., 2009). In contrast, Sirt1 activation was protective in an ALS and other Huntington’s disease models (Kim et al., 2007; Jiang et al., 2011; Watanabe et al., 2014). These conflicting data in numerous brain disease models led us to examine whether blocking Sirt1 activity will influence epilepsy development after a proepileptogenic insult.

## Materials and Methods

### Animals

All experiments were approved by the University of California, Irvine, Institutional Animal Care and Use Committee and conformed to National Institutes of Health guidelines. Adult male, 2 month old Sprague-Dawley rats (Harlan; RRID:RGD_5508397) were housed under a 12 h light/dark cycle, with *ad libitum* access to food and water. We made all efforts to minimize the number and suffering of the animals.

### Surgery

We anesthetized rats at an average of 300 g [two control (CTRL) groups, *n* = 5/group; two kainic acid-induced SE (KA-SE) groups, *n* = 14/group with inhalation of 4% isoflurane]. Rats were shaved and placed into a stereotaxic frame, and their eyes were hydrated. We treated the skin with iodine and ethanol then made a midline scalp incision to expose the skull. We cleaned the skull and drilled holes based on coordinates. We positioned bilateral infusion cannulae on the cortical surface (distance from bregma: −1.0 mm posterior; ±1.5 mm lateral) directly above the lateral ventricles, using the coordinates of Paxinos et al. (1985). We implanted bipolar electrodes (Plastics One) bilaterally into the hippocampus (distance from bregma: anteroposterior, 3.3 mm; lateral, 2.3 mm; ventral, −2.8). For the tethered system, we secured two reference skull screw and skull screws to anchor the head cap. We encased the electrodes, cannulae, and screws in dental cement. For the remote system from Data Science International (DSI), we created a subcutaneous pocket using scissors with a blunt end in the flank region and inserted an implantable small animal CNS telemetry probe (model F40-EET, DSI). The incision was sutured closed. Rats received 5 ml of 0.9% saline via subcutaneous injection to rehydrate and aid in recovery from surgery. We monitored the rats and allowed them 5–7 d postsurgical recovery before any further experimental procedures were conducted.

### Generation of status epilepticus

We induced SE, as previously described (McClelland et al., 2011; Brennan et al., 2016), following the procedure devised by Hellier and Dudek (2005). Briefly, rats were intraperitoneally injected repeatedly with 5 mg/kg kainic acid (catalog #ab120100, Abcam) to reach and maintain SE for 3 h while controls received saline. We continuously monitored and scored seizures using the Racine (1972) scale. After 3 h of SE, KA-SE rats received 8 mg/kg dose of diazepam (Patterson Veterinary) to terminate SE. Controls received saline. We monitored the behavior of the rats for overt seizure activity and gave another diazepam dose if necessary. We gave a subcutaneous injection of 5 ml of 0.9% saline for rehydration and provided a soft food diet for 3 d.

### Intracerebroventricular injection

Immediately following the termination of the SE, we anesthetized the rats with 4% isoflurane and maintained them with 2.5%, then we infused either EX-527 (catalog #E7034, Sigma-Aldrich) or vehicle (VEH) into the lateral ventricle. The cannula needle was lowered so that the tip was within the lateral ventricle. We then infused 5 µg/ventricle (4.6 µl at 1.09 µg/µl) of EX-527 or 4.6 µl of vehicle (1.8% EtOH and 0.5% DMSO in saline) at a rate of 0.5 µl/min.

### Digital video–electroencephalogram recording and analyses

Following SE, we began video-electroencephalogram (EEG) monitoring. EEG recording were synchronized to video and conducted for a period of up to 2 months. Two experienced investigators who were blind to group identity visually scanned the coded EEGs for seizures (Dube et al., 2010). We analyzed the concurrent video recordings for behavioral manifestations of the apparent seizure. Only events with EEG and behavioral changes and that lasted >10 s were classified as seizures. We evaluated typical behaviors associated with limbic-onset seizures, including sudden cessation of activity, facial automatisms, head bobbing, and prolonged immobility with staring. These progressed to alternating or bilateral clonus, rearing, and falling (Racine, 1972). Rats were considered epileptic if they had at least one documented seizure as defined above. As a measure of network hyperexcitability, we recorded interictal spikes and spike series and quantified them for a subset of rats (*n* = 6 per group) over 3 consecutive days (days 28–30 of the recordings; White et al., 2010). Criteria for spikes were 20–70 ms, and amplitude at least two times baseline. Concurrent video monitoring was used to exclude chewing movement and electrical noise (White et al., 2006). We used a tethered system (PowerLab data acquisition hardware, Bio Amplifiers, and Labchart7 software, AD Instruments) and an implantable telemetry system (with Neuroscore3.0 software, DSI) for EEG acquisition, and we used the same software for seizure detection and analysis. In *post hoc* analyses, there were no differences between the tethered and implantable systems in any parameter.

### Quantitative reverse transcription-PCR

We anesthetized and decapitated the rats and then dissected out their hippocampi using prechilled RNase-free instruments. Hippocampi were frozen immediately and stored at −80°C until use. We isolated total RNA from the left hippocampus using the *mir*Vana miRNA Isolation Kit with phenol (catalog #AM1560, Ambion) according to the manufacturer instructions. We quantified and analyzed RNA purity by nanodrop. We excluded samples if RNA with low purity. We synthesized cDNA using a combination of random hexamers and anchored oligodT primers (Transcriptor First Strand cDNA Synthesis Kit, Roche) or with specific hairpin stem loop primers for microRNA (miRNA) analysis. cDNA was prepared using an Eppendorf Thermocycler on a standard heating–cooling cycle. cDNA was diluted 10-fold and stored at 4°C for up to 1 month. We performed quantitative PCR (qPCR) using SYBR Green chemistry (Roche) on a Roche Lightcycler 96. We analyzed the relative quantitative amounts using the cycle threshold method (2^-ΔΔCt). We determined the levels of mature miRNA-124 using the miR124a TaqMan MicroRNA Assay (catalog #4427975, ThermoFisher Scientific) primers designed for the 3' arm. Levels of inflammatory mediators were determined using primer sequences reported in Table 1. GAPDH and 14-3-3 ζ were used for normalization (Table 1). Minus-reverse transcription and nontemplate controls were routinely used to eliminate the possibility of genomic contamination or false-positive analyses.

**Table 1:** Primers used for qPCR

mRNA	Forward primer	Reverse primer
COX-2	TGGTGCCGGGTCTGATGATG	GCAATGCGGTTCTGATACTG
CCL3	GGGTGTCATTTTCCTGACCAAGAGAAACCG	GCTGCCTCTAATCTCAGGCATTTAGTTCCAG
IL-1β	GTGAAATAGCAGCTTTCGACAGTGAGGAG	GTGAGATTTGAAGCTGGATGCTCTCATCTG
TNF-α	CCCAGACCCTCACACTCAGAT	TTGTCCCTTGAAGAGAACCTG
GAPDH	ATGCCATCACTGCCACTCAGA	ACCAGTGGATGCAGGGATGAT
14-3-3 ζ	ACCGTTACTTGGCCGAGGTT	ACCGTTACTTGGCCGAGGTT

### Chromatin immunoprecipitation

We anesthetized, and decapitated KA-SE rats 1 h after SE termination, and we isolated the hippocampi. The right hippocampus was cross-linked and homogenized, and the nuclei were harvested by centrifugation. Nuclei were sonicated and precleared with Protein-A/G (Santa Cruz Biotechnology). We then immunoprecipitated samples with 10 µg of either control nonimmune serum (IgG; catalog #2729S, Cell Signaling Technology; RRID:AB_1031062) or anti-Sirt1 (H-300; catalog #sc-15404, Santa Cruz Biotechnology; RRID:AB_2188346) overnight at 4°C. We precleared protein A/G beads (catalog #sc-2003, Santa Cruz Biotechnology) and added them to the lysate for 2 h. We washed beads to remove nonspecifically bound protein and performed SDS elution. Eluates were reverse cross-linked, and the bound DNA was purified using the QIAquick MinElute PCR purification kit (catalog #28104, Qiagen). We performed qPCR amplification using SYBR Green chemistry (Roche). The *MIR124-1* primer sequence (5' to 3') was as follows: forward, TCCTTAGCTCATCGCGTTCC; reverse, AGCCCCATTCTTGGCATTCA in Sirt1 binding region. We report antibody binding to the gene as the percentage of input. We calculated the percentage of input as follows. Triplicate Ct (cycle threshold) values for each sample were averaged for input, Sirt1 chromatin immunoprecipitation (ChIP) and IgG. Adjusted input values for each sample were subtracted from the averaged Sirt1 ChIP and IgG values. We then performed a ΔΔCt calculation multiplied by 100 to obtain the final percentage of input in Excel, as follows: (power (efficiency of primer −(IP − adjusted input))*100).

### Immunohistochemistry

At the end of 2 months of video EEG recording, we killed the rats and rapidly removed their brains, which were bisected sagittally. We postfixed hemibrains in 4% paraformaldehyde at 4°C for 24 h, cryoprotected them in 30% sucrose for a minimum of 24 h, then flash froze them in isopentane and stored them at −80°C. Then we froze the hemibrains to a chuck using OCT and sliced them coronally at −20°C by cryostat (model CM1900, Leica) into 25 µm sections. Serial sections were collected in cryoprotectant (30% ethylene glycol, 30% sucrose, 0.1 m PB) and stored at −20°C. We stained one serial section from each rat for NeuN (catalog #MAB377, Millipore; RRID:AB_2298772). We quenched sections with 0.3% hydrogen peroxide in 10% methanol for 10 min and blocked in 10% normal goat serum (NGS; r527-4001, Sigma-Aldrich) in 0.01 m PBS with 0.03% Triton-X (PBS-T) for 1 h. Then we incubated sections in primary antibody against NeuN at 1:1000 in 4% NGS in PBS-T overnight at 4°C followed by incubation with biotinylated mouse secondary antibody at 1:400 (catalog #BA-9200, Vector Laboratories; RRID:AB_2336171) for 1 h. Subsequently, incubation in Vectastain ABC HPR kit (catalog #PK-6100, Vector Laboratories) was performed for 1 h. We visualized sections using the directions on the DAB Kit (catalog #SK-4100, Vector Laboratories). Sections were mounted on slides and coverslipped using Permount Mounting Medium (catalog #SP15, Fisher Chemicals). An investigator blinded to treatment group took images of three sections per animal with a 10× objective on a Nikon Eclipse E400 microscope. Using ImageJ software, we drew an 810 × 810 µm box around the CA3 pyramidal cell layer and around the hilus excluding the granule cell layer. We then counted by hand the NeuN-positive cells within these boundaries.

### Analyses and statistical considerations

All assessments and analyses were conducted without the knowledge of experimental group. Group sizes were determined a priori during the experimental design phase based on power analyses. Statistical analyses were performed using GraphPad Prism (RRID:SCR_002798) software, and all data are expressed as the mean ± SE, unless otherwise stated. Differences between the two groups were evaluated using unpaired Student’s *t* test, and comparisons of multiple factors were evaluated using two-way ANOVA. Error bars represent the SEM. Outliers were excluded using the GraphPad Prism ROUT test for outliers (Table 2).

**Table 2:** Statistics

Figure	Data structure	Statistical test	Power/significance level
1B	Normal distribution	Unpaired *t* test	0.0317
2A	Normal distribution	Two-way ANOVA, Tukey’s test	Drug, 0.0032 KA-SE, 0.8793 Interaction, 0.064
2B	Normal distribution	Two-way ANOVA, Bonferroni’s test	Drug, 0.02080 KA-SE, 0.0705 Interaction, 0.0625
3A	Nonparametric	Mann–Whitney test	0.2831
3B	Nonparametric	Mann–Whitney test	0.0990
3C	Nonparametric	Mann–Whitney test	0.3256
3D	Nonparametric	Mann–Whitney test	0.1429
3E	Nonparametric	Mann–Whitney test	0.7533
3G	Nonparametric	Mann–Whitney test	0.9004
3H	Nonparametric	Mann–Whitney test	0.4242
4A	Normal distribution	Two-way ANOVA, Tukey’s test	Drug, 0.4538 KA-SE, <0.0001 Interaction, 0.5467
4B	Normal distribution	Two-way ANOVA, Tukey’s test	Drug, 0.3588 KA-SE, 0.0002 Interaction, 0.1033
4C	Normal distribution	Two-way ANOVA, Tukey’s test	Drug, 0.3638 KA-SE, 0.1229 Interaction, 0.0579
4D	Normal distribution	Two-way ANOVA, Tukey’s test	Drug, 0.1461 KA-SE, 0.8726 Interaction, 0.0198

## Results

### Sirt1 activity increases rapidly after kainic acid-induced SE

**Figure 1.**
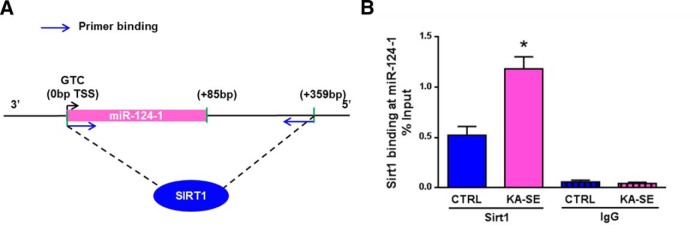
Sirt1 activity increases after KA-SE in adult rats. ChIP was used to assess the binding of Sirt1 to chromatin, an indicator of its deacetylase activity. Hippocampi from KA-SE and control rats were obtained 1 h after KA-SE termination. We assessed specifically Sirt1 binding to the miR-124-1 gene promoter. ***A***, Schematic of Sirt1 binding to miR-124-1 gene promoter and the locations of primer sequences. ***B***, Sirt1 binding to the promoter of the miR-124-1 gene in KA-SE rats is significantly higher compared with that in CTRL rats (Student’s *t* test, *p < 0.05). Binding is shown as the percentage of input. The specificity of this binding is demonstrated by comparing it with the nonspecific binding of IgG to chromatin. TSS, Transcription start site; blue arrow, primer binding site (*n* = 4-6/group).

Sirt1 activity depends on NAD^+^ (Blander and Guarente, 2004). The NAD^+^/NADH ratio is increased during metabolically demanding events (Srivastava, 2016). Therefore, Sirt1 activity is typically enhanced during periods of the mismatch of supply and demand (Blander and Guarente, 2004; Herskovits and Guarente, 2014). Here we examined whether KA-SE sustained bursts of neuronal activity that require large amounts of neuronal energy (Duffy et al., 1975; Fujikawa et al., 1988; Carmody and Brennan, 2010), leading to increased Sirt1 activity. An established action of Sirt1 is to deacetylate histones within the chromatin, so we used ChIP to assess Sirt1 binding to a region of its previously established target gene (Brennan et al., 2016) miRNA 124-1 (*MIR124-1*; Fig. 1*A*). Three genes (*MIR124-1*, *MIR124-2*, and *MIR124-3*) code for pre-miRNAs that are all cleaved into mature miR-124, but only *MIR124-1* is modulated by Sirt1 binding (Brennan et al., 2016). In hippocampus of KA-SE rats, Sirt1 binding to the *MIR124-1* gene doubled at 1 h after SE termination compared with levels of Sirt1 binding in rats not experiencing KA-SE (Student’s *t* test, *p* < 0.05; *n* = 4-6/group; Fig. 1*B*). Nonspecific IgG binding was minimal and similar between groups (Fig. 1*B*).

### Administration of EX-527 blocks KA-SE-induced Sirt1 activation

**Figure 2.**
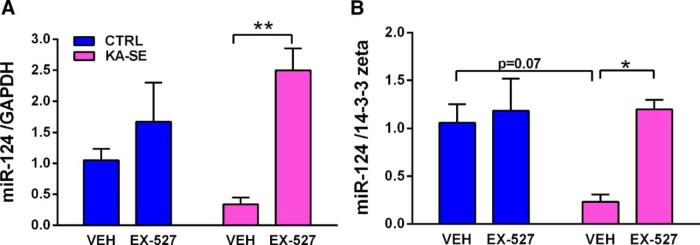
The Sirt1 inhibitor EX-527 blocks Sirt1 activity after KA-SE. ***A***, ***B***, Hippocampal mature miR-124 levels were measured by qPCR at 4 h after KA-SE termination in rats infused with vehicle or the Sirt1 inhibitor (EX-527) and normalized to GAPDH levels (***A***) or 14-3-3 ζ levels (***B***). ***A***, KA-SE reduced miR-124 levels and EX-527 restored miR-124 levels (two-way ANOVA; main effect of inhibitor, *p* < 0.01; *post hoc*, **p < 0.01; *n* = 4/group). ***B***, KA-SE reduced miR-124 levels and EX-527 restored miR-124 levels when normalized to 14-3-3 ζ (two-way ANOVA; main effect of inhibitor, *p* < 0.05; *post hoc* , *p < 0.05; *n* = 4/group).

EX-527 blocks Sirt1 function by occupying the NAD^+^ binding site of Sirt1 (Gertz et al., 2013). Here, rats were infused intracerebroventricularly with 5 µg/hemisphere EX-527 or vehicle. To test whether EX-527 effectively blocked Sirt1 activity, we capitalized on the established role of Sirt1 in mediating KA-SE-induced reduction of miR-124 (Brennan et al., 2016). We measured hippocampal levels of mature miR-124 at 4 h after KA-SE termination. We found that miR-124 levels were reduced in KA-SE rats compared with control rats (Fig. 2*A*,*B*). Administration of EX-527 prevented this reduction (and tended to increase levels in control rats). Indeed, miR-124 levels in treated KA-SE rats were higher than in those in controls normalized to GAPDH (main effect of inhibitor: *F*_(1,12)_ = 13, *p* < 0.01; trend for interaction: *F*_(1,12)_ = 4.16, *p* = 0.064; *post hoc*: KA-VEH vs KA-EX-527, *p* < 0.01; *n* = 4/group) or 14-3-3 ζ (main effect of inhibitor: *F*_(1,12)_ = 7.08, *p* < 0.05; trend for interaction: *F*_(1,12)_ = 4.22, *p* = 0.065; trend for main effect of KA-SE: *F*_(1,12)_ = 3.94, *p* < 0.07; *post hoc*: KA-VEH vs KA-EX-527, *p* < 0.01; CTRL-EX-527 vs KA-VEH: *p* < 0.01; trend CTRL-VEH vs KA-VEH, *p* = 0.07; *n* = 4/group).

### Effect of Sirt1 inhibition on epilepsy development

**Figure 3.**
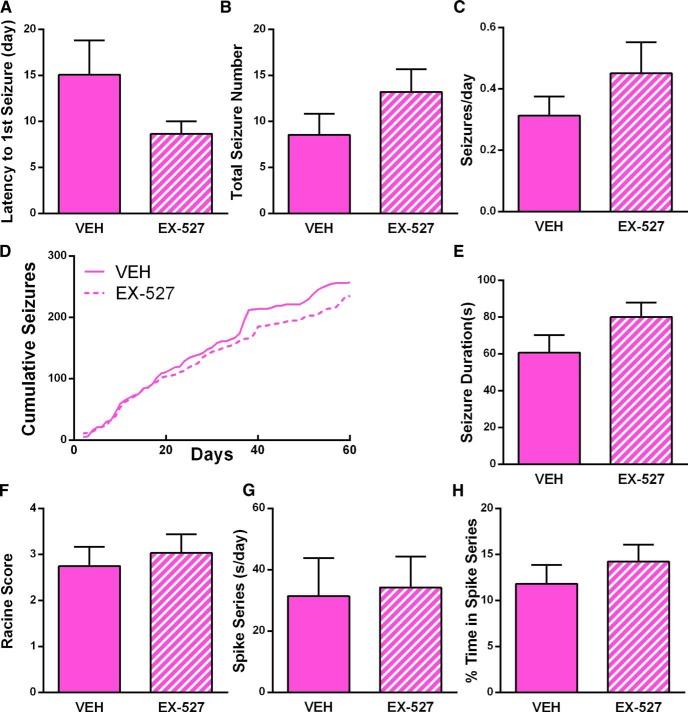
Sirt1 inhibition does not prevent the development of epilepsy. Continuous digital video–EEG for 2 months was used to examine for spontaneous seizures in KA-SE rats infused with either vehicle (VEH) or Sirt1 inhibitor (EX-527) (n = 14/group). Most rats (26 of 28) developed spontaneous seizures (epilepsy) independent of treatment. ***A***, The latency to the onset of the first seizure was not different between VEH- and EX-527-infused rats. ***B***, The total number of seizures was not different between VEH- and EX-527-infused rats. ***C***, The average number of seizures per day was not different between VEH- and EX-527-infused rats. ***D***, Cumulative seizure numbers did not distinguish between groups. ***E***, Median seizure duration did not differ between VEH- and EX-527-infused rats. ***F***, Median seizure severity (using the Racine scale) was similar between VEH and EX-527 rats. ***G***, Average frequency of interictal spike series per day did not distinguish the EX-527-treated and VEH-treated groups. ***H***, The percentage of time spent in spike series after KA-SE was not different with EX-527 treatment (for spike series analysis: *n* = 6/group).

We subjected KA-SE and control rats to continuous digital video–EEG for 2 months. To examine whether Sirt1 inhibition blocks epileptogenesis, a subset of KA-SE rats (*n* = 14) was administered the antagonist EX-527 immediately after the KA-SE and compared with those given vehicle (*n* = 14). We evaluated the presence of spontaneous seizures, average number of seizures per day, latency to the onset of the first seizure, median seizure duration, and median seizure severity as determined by the Racine scale. The large majority of KA-SE rats (26 of 28; 93%) developed spontaneous seizures (epilepsy). Mean latency to the first seizure for vehicle-infused rats was 15 d in EX-527-infused rats was 8.6 d (Fig. 3*A*). The average total seizure number was 8.5 in KA-SE rats infused with vehicle and 13 in KA-SE rats infused with EX-527 (Fig. 3*B*). The average numbers of seizures per day did not distinguish the groups (0.31 and 0.45 seizure/day, respectively, in VEH- and EX-527-infused rats; Fig. 3*C*). The cumulative number of seizures for all 14 rats per experimental group was not different between the control and KA-SE groups (Fig. 3*D*), suggesting that the progression of epileptogenesis was similar. Vehicle- and EX-527-infused rats had similar median seizure durations of 61 and 80 s, respectively (Fig. 3*E*). The median seizure severities, as determined using the Racine scale, were 2.8 and 3, respectively, in vehicle- and EX-527-infused KA-SE rats (Fig. 3*F*). As a measure of network hyperexcitability, we analyzed a subset of the rats for the presence and frequency of interictal spike series (*n* = 6 rats/group). The average number of spike series per day and the percentage of time spent in spiking were similar between the groups during days 28–30 of the recordings (Fig. 3*G*,*H*). Together, these data indicate that Sirt1 inhibitor administration immediately after the insult, although effective in blocking Sirt1 function, did not influence the development of a hyperexcitable network or of epilepsy within the 2 month time frame studied here.

### Effect of Sirt1 inhibition on inflammation

**Figure 4.**
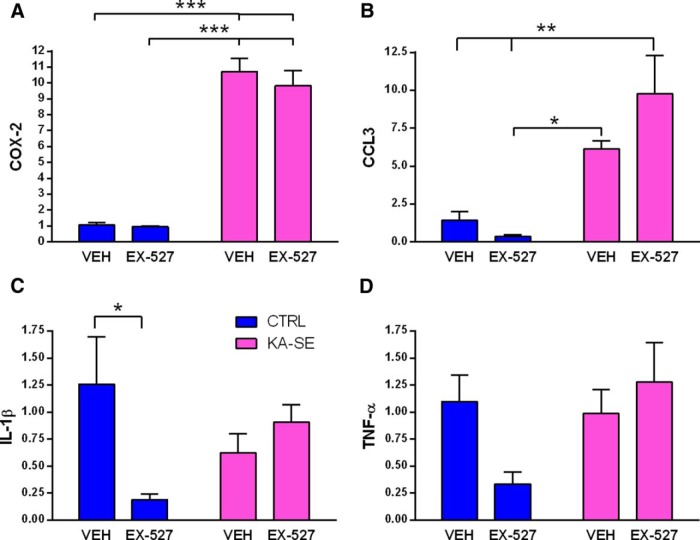
Sirt1 inhibition did not abrogate acute inflammation induced by KA-SE. Inflammatory markers in rat hippocampus were measured using qPCR at 4 h after KA-SE termination. ***A***, COX-2 levels were strikingly increased by KA-SE; these levels were not affected by Sirt1 inhibitor EX-527 (two-way ANOVA; KA-SE main effect: *F*_(1,12)_ = 204, *p* < 0.001; *post hoc*, ***p < 0.001). ***B***, CCL3 mRNA levels were increased in the KA-SE groups, but EX-527 had no effect (KA-SE main effect: *F*_(1,12)_ = 28, *p* < 0.001; *post hoc*, **p < 0.01,*p < 0.05). ***C***, IL-1β levels were significantly reduced by the administration of EX-527 to control rats. The inhibitor did not influence cytokine IL-1β levels in KA-SE rats (two-way ANOVA; interaction of KA-SE and inhibitor: *F*_(1,12)_ = 7, *p* < 0.05; *post hoc*, *p < 0.05). ***D***, No significant changes in TNF-α were observed after EX-527 infusion (two-way ANOVA; trend for interaction of KA-SE and drug: *F*_(1,12)_ = 4.4, *p* = 0.06; *n* = 4/group).

Inflammatory processes are rapidly activated by SE, including KA-SE, and have been shown to contribute to epileptogenesis (Vezzani et al., 2011; Brennan et al., 2016). Therefore, we sought to examine the potential effect of blocking Sirt1 on KA-SE-provoked activation of specific inflammatory pathways. We analyzed mRNA expression levels of several inflammatory mediators at 4 h following SE termination, a time where many of these pathways are activated (Jiang et al., 2015; Patterson et al., 2015). We found a significant increase in cyclooxygenase-2 (COX-2) expression in the hippocampus in both KA-SE treatment groups, which is consistent with prior reports (KA-SE main effect: *F*_(1,12)_ = 204, *p* < 0.001; *post hoc* for KA-VEH vs CTRL-VEH, KA-VEH vs CTRL-EX-527, KA-EX-527 vs CTRL-EX-527, CTRL-VEH vs KA-EX-527, *p* < 0.001; *n* = 4/group; Gorter et al., 2006; Jiang et al., 2015). Notably, EX-527 had no effect on COX-2 mRNA levels (Fig. 4*A*). The chemokine CCL3, which has been shown to be augmented after status epilepticus (Arisi et al., 2015), was upregulated by KA-SE (KA-SE main effect: *F*_(1,12)_ = 28, *p* < 0.001; *post hoc* CTRL-VEH vs KA-EX-527, CTRL-EX-527 vs KA-EX-527, *p* < 0.01; CTRL-EX-527 vs KA-VEH, *p* < 0.05; Fig. 4*B*). We interrogated the IL-1β pathway because of its established role in epileptogenesis (Noe et al., 2013). We found that IL-1β was decreased in the control rats treated with EX-527 compared with controls treated with vehicle (interaction of KA and inhibitor: *F*_(1,12)_ = 7, *p* < 0.05; *post hoc* CTRL-VEH vs CTRL-EX-527, *p* < 0.05; Fig. 4*C*). We found little activation of IL-1β by KA-SE at the 4 h time point, which may be too early to see activation (Vezzani et al., 2011). We examined tumor necrosis factor-α (TNF-α) and found no significant effect of KA-SE. TNF-α expression in EX-527-treated controls tended to decrease compared with vehicle-treated controls (trend for interaction of KA-SE and inhibitor: *F*_(1,12)_ = 4.4, *p* = 0.06; Fig. 4*D*). In summary, KA-SE activated a number of inflammatory pathways; EX-527, while attenuating IL-1β and TNF-α in control rats, had no effect on KA-SE-induced activation of these inflammatory mediators.

### Effect of Sirt1 inhibition on KA-SE-induced cell loss

**Figure 5.**
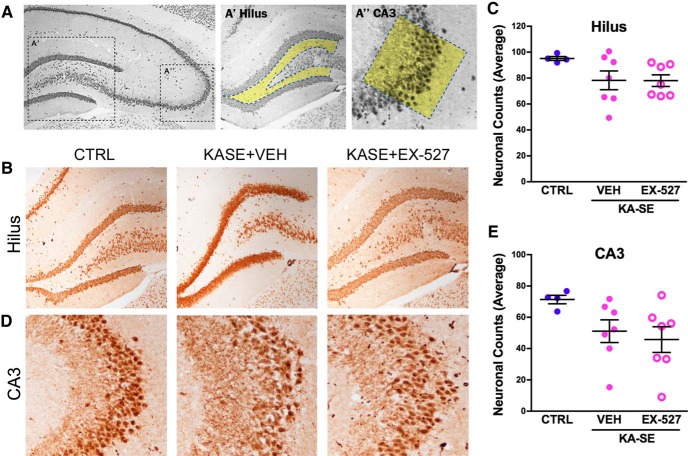
Cell loss after KA-SE was modest and was not affected by Sirt1 inhibition. NeuN straining was use to visualize neuronal dropout in the hippocampus 2 months after kainic acid–induced SE. ***A***, Schematic of location for neuronal counts in the hilus and CA3 regions of the hippocampus. ***B***, Representative images of average neuronal counts in the hilus region from CTRL and KA-SE rats treated with VEH or EX-527. ***C***, Quantification of neuronal dropout in the hilus shows a small, insignificant loss of neurons in the hilus. ***D***, Representative images of average neuronal counts in the CA3 region from CTRL and KA-SE rats treated with VEH or EX-527. ***E***, Quantification of neurons in the CA3 region shows a small but insignificant loss of neurons in the CA3. (Controls, *n* = 4; KA-SE rats, *n* = 7/group).

Focusing on the hippocampal formation, NeuN-positive cells were counted 2 months after KA-SE (Fig. 5*A*). Neuronal counts were modestly lower in the hilus region (Fig. 5*B*,*C*) and the pyramidal cell layer of CA3 (Fig. 5*D*,*E*) of KA-SE rats compared with control rats. There were no differences between the KA-SE groups that received vehicle or EX-527. These data suggest that the lack of an antiepileptogenic effect of Sirt1 blockade was not a result of the inability of the inhibitor to overcome KA-SE-induced massive cell loss.

## Discussion

In the current studies, we found that KA-SE induces Sirt1 activation in the hippocampus. We successfully blocked Sirt1 activity *post hoc* in rats experiencing KA-SE using EX-527. Unexpectedly, we found that blocking Sirt1 activity immediately after SE neither prevented nor exacerbated any measure of epileptogenesis. These data suggest that epileptogenesis following status epilepticus can proceed via pathways that are independent of Sirt1 activity.

There is evidence for Sirt1 activation in epilepsy. Sirt1 was upregulated in epilepsy patients (Chen et al., 2013) and increased in rat models of epilepsy within 1 h (Wang et al., 2015; Brennan et al., 2016). We found the increase in the histone deacetylase activity of Sirt1 already at 1 h following KA-SE in the current study. The time course of Sirt1 activation is short, providing support for our approach of short-term blockade of Sirt1. Interestingly, Wang et al., 2016 reported a reduction of Sirt1 later than 24 h after SE (Wang et al., 2016). Our previous data indicated that blocking Sirt1 with EX-527 immediately following KA-SE *in vitro* restores the levels of a target gene of Sirt1, miR-124, and this effect lasted for at least 48 h (Brennan et al., 2016). Therefore, here we aimed to block the activity of Sirt1 within the first hours following KA-SE.

We confirmed that the dose of EX-527 used here was effective at blocking the histone deacetylase activity of Sirt1. Yet, blocking Sirt1 actions neither prevented nor exacerbated any measure of network hyperexcitability and epileptogenesis. There are several possible reasons for these findings. First, it might be possible that EX-527 inhibition might be nonspecific: EX-527 is ∼500 fold more specific to Sirt1 than to Sirt2 and Sirt3 (Selleck Chemicals, http://www.selleckchem.com/products/EX-527.html). Considering that we administered 10 µg of the compound over 20 min into a rapidly following CSF, it is not very likely that EX-527 reached the IC_50_ of Sirt2 or Sirt3. Second, within the pathway we targeted, Sirt1 regulates miR-124, preventing its repression following KA-SE. This miRNA has dual and opposing effects on epileptogenesis. On one hand, miR-124 blocks proepileptogenic molecular cascades; on the other hand, the same microRNA exacerbates inflammation (Brennan et al., 2016). Thus, blocking Sirt1 would counteract both the proepileptogenic and antiepileptogenic actions of miR-124.

In addition, Sirt1 acts as a protein and histone deacetylase, thus directly or epigenetically regulating many cell processes such as metabolism, apoptosis, autophagy, and mitochondrial function. These diverse actions might exert a negative or positive effect on epileptogenesis. For example, Sirt1 activates peroxisome proliferator-activated receptor gamma coactivator 1-α, which ameliorates mitochondria dysfunction and activates ROS-detoxifying enzymes (Wang et al., 2015). Sirt1 activation of forkhead box class O counters cell stress and promotes cell survival (Giannakou and Partridge, 2004). Sirt1 inactivates p53 and nuclear factor-κB subunit p65/RelA, which are involved in cell death (Soengas et al., 1999). In addition, Sirt1 can modulate DNA methyltransferase 1 activity, leading to transcriptional repression (Peng et al., 2011), which has another multitude of effects.

Sirt1 interacts with poly(ADP-ribose) polymerase-1 (PARP-1) through NAD^+^ (Min et al., 2013; Imai and Guarente, 2014). PARP-1 activity competes for the same coactivator, NAD^+^. PARP-1 depletion of NAD^+^ leads to energy failure and cell death (Oliver et al., 1999; Hassa and Hottiger, 2002; Wang et al., 2013). Sirt1 suppresses PARP-1 gene transcription (Rajamohan et al., 2009). Therefore, Sirt1 and PARP-1 activities are inversely related (Walko et al., 2014). PARP-1 inhibition prevents cell death in an epilepsy model (Wang et al., 2007). In our model, Sirt1 inhibition might increase PARP-1 activation, which could contribute to NAD^+^ depletion and cell death. Notably, we did not find augmented cell death following Sirt1 blockade in the current studies (Fig. 5).

Finally, blocking Sirt1 is expected to have divergent actions on inflammation, mediated by distinct mechanisms. By preventing KA-SE-induced repression of miR-124, blocking Sirt1 should either reduce or increase microglia activation (Brennan et al., 2016). Sirt1 also has direct effects on inflammation (Cho et al., 2015).

In summary, Sirt1 is sensitive to metabolic stress signals within the cell, and is rapidly and potently upregulated early in the process of epileptogenesis. Whereas it is a theoretically attractive target for disease modification, the current work indicates that Sirt1 may not be the ideal target for blocking epileptogenesis. In part, this may be a result of the multitude of effects of Sirt1 on numerous cellular and molecular processes.

## Synthesis

Reviewing Editor: Alfonso Represa, INSERM Decisions are customarily a result of the Reviewing Editor and the peer reviewers coming together and discussing their recommendations until a consensus is reached. When revisions are invited, a fact-based synthesis statement explaining their decision and outlining what is needed to prepare a revision will be listed below. The following reviewer(s) agreed to reveal their identity: Matthew Walker

Dear authors,

Your manuscript has been reviewed by to referees that agree on the interest and quality of the research proposed and both ask for clarification of number of points (described before). We think that they will help improving the quality of the manuscript .

1) It would be helpful in this paper if the various roles of Sirt1 are discussed in more detail upfront in the introduction, so that the reader is aware of the controversy in the literature. This is addressed in the final paragraph but could be more explicit (including the recent epilepsy literature).

2) One major criticism could be leveled at the specificity of EX-527. In total 10ug are being injected into the rats' ventricles. Since total rat CSF volume is approximately 100uL then this is equivalent to a potential peak concentration of 100g/L. Since the MW is ~250, then the peak concentration would be about 400mM above the IC50 for an action on Sirt2 and Sirt3. Since the Sirts can have opposing effects this is important - it would be nice to know if a lower concentration of Ex-527 may have been effective or at least have some discussion concerning specificity!

3) The seizure counts are convincing. However, there is a rather specific definition of seizures used. It is likely that no effect on the frequency of seizure events over 20s would translate to other definition of seizure events (eg >10s). However, since there is continuous EEG it would be nice to know whether there is any effect on EEG coastline and/or briefer events.

4) In an abstract authors state following: “Post-hoc infusion of the Sirt1 inhibitor prevented Sirt1-mediated-repression of target genes.” However, in the results paragraph authors present only results for miR-124. It would be nice to see if other targets of Sirt1 were altered (upregulated as compared to veh-treated) after inhibition.

5) Authors assessed hippocampal levels of miR-124 at 4h post-SE. How long Sirt1 inhibition restore miR-124 levels?

6) Authors should address the KA-SE-induced cell loss with (for example) quantitative method. Now, in the text there is “no observable difference”, and still in the figure there is different amounts of arrows in panels. This is confusing. Also, the Fig5 is not suitable for showing any changes in cell loss. Many panels are not good quality presentations. Further, authors do not present or discuss results related to inhibitory neuron loss after KA-SE (references as Buckmaster and Dudek, 1997, Kuruba et al 2011). Results presenting cell loss should be either removed or they need to be clarified and (preferably) assessed with quantitative method. However, removing will definitely impair the impact of the paper.

7) Generation of status epilepticus and administration of EX-527: Please provide the time point when Diazepam was administered. Did controls also receive Diazepam? Was EX-527 administered immediately after Diazepam? If not, how long time there was between these two drugs? How authors assessed the effect of Diazepam to results obtained?

8) Materials and methods paragraph have to be rewritten. Please provide following information:

a. Surgery: Please include weight of the rats when experiments were started. Also, weight of the rats during follow-up and recovery should be noted as well as exclusion criteria.

b. Administration of EX-527: Please indicate which anesthesia was used. Also, please provide what kind of post-operative care rats obtained after SE.

c. EEG analysis: Please provide which systems were used and manufacturers.

d. qPCR: Indicate how rats were killed, time point after SE and how fast tissue piece was flash frozen. Was left or right hemisphere used for this analysis? Please add cut-off values for Nanodrop measurements. Provide all reagent or kit catalogue numbers. How miR-124 levels were normalized? Is miR-124 primers used here for mature miRNA?

e. ChIP: Indicate how rats were killed, time point after SE and how fast tissue piece was flash frozen. Was left or right hemisphere used for this analysis? Provide all reagent or kit catalogue numbers, temperatures in with experiments were done and concentrations used.

f. Neuroanatomical assessment: How long cryoprotection was used? Please provide detailed protocol for cryostat slicing. Please see comment 3.

9) Figures

a. Fig1: How % Input was calculated. Is <1% change biologically relevant amount? How EX-527 changes this binding?

b: Fig2: Is this for mature miRNA? Which arm? Is there difference between vehicle treated rats (Control vs SE)?

c. I cannot understand figure3 at all. How latency to the first seizure can be 10-15 days but in the panel D, the lines start close to day 0? Also there is total number of seizures in the panel B, 10-15 seizures, and in panel D it is hundreds. Moreover, it looks like in panels B and C that veh-rats had less seizures, but in the panel D there is more seizures presented for veh-treated rats.

d. Fig4: How authors explain IL-1beta huge difference in EX-527 treated controls as compared veh-controls?

10) Please indicate if data was normally distributing as parametric tests were used. Also because ANOVA was used, was the test of homogeneity of variances done, was there independence of observations and no significant outliers?

11) Minor comments: Please check the writing style for gene names and proteins.
